# Large genetic yield potential and genetic yield gap estimated for wheat in Europe

**DOI:** 10.1016/j.gfs.2019.100340

**Published:** 2020-03

**Authors:** Nimai Senapati, Mikhail A. Semenov

**Affiliations:** Department of Plant Sciences, Rothamsted Research, West Common, Harpenden, Hertfordshire, AL5 2JQ, United Kingdom

**Keywords:** Crop modelling, Cultivar trait optimisation, Ideotype design, Sirius wheat model, Wheat improvement

## Abstract

Improving yield potential and closing the yield gap are important to achieve global food security. Europe is the largest wheat producer, delivering about 35% of wheat globally, but European wheat's yield potential from genetic improvements is as yet unknown. We estimated wheat ‘genetic yield potential’, i.e. the yield of optimal or ideal genotypes in a target environment, across major wheat growing regions in Europe by designing *in silico* ideotypes. These ideotypes were optimised for current climatic conditions and based on optimal physiology, constrained by available genetic variation in target traits. A ‘genetic yield gap’ in a location was estimated as the difference between the yield potential of the optimal ideotype compared with a current, well-adapted cultivar. A large mean genetic yield potential (11–13 t ha^−1^) and genetic yield gap (3.5–5.2 t ha^−1^) were estimated under rainfed conditions in Europe. In other words, despite intensive wheat breeding efforts, current local cultivars were found to be far from their optimum, meaning that a large genetic yield gap still exists in European wheat. Heat and drought tolerance around flowering, optimal canopy structure and phenology, improved root water uptake and reduced leaf senescence under drought were identified as key traits for improvement. Closing this unexploited genetic yield gap in Europe through crop improvements and genetic adaptations could contribute towards global food security.

## Highlights

• Large wheat genetic yield potential (11–13 t ha^−1^) and genetic yield gap (3.5–5.2 t ha^−1^) estimated by ideotypes for Europe.
• The wheat genetic yield potential has still to be fully utilised by breeders in Europe.
• Closing the unexploited genetic yield gap in Europe would contribute to global food security.

## 1 Introduction

Ensuring global food security, while protecting the environment, non-agricultural lands and biodiversity, is the single greatest scientific challenge facing humankind (Cassman, 2012). It has been predicted that food production needs to increase by about 70% between 2007 and 2050 to feed an estimated >9 billion people (FAO, 2009; UN, 2017). On the other hand, food security is under threat from ongoing climate change, plateauing of crop yield in many regions and diminishing natural resources (FAO, 2018). Increasing crop yield potential and closing the yield gap are two important aspects of the solutions proposed to achieve global food security in a sustainable manner with minimum environmental footprints (Godfray et al., 2010; Foley et al., 2011).

The yield gap of a crop in a certain location is defined as the difference between yield of a present, well-adapted cultivar (genotype) under optimal crop management (irrigated/rainfed) and the average actual yield achieved by farmers under dominant management practices (Lobell et al., 2009; van Ittersum et al., 2013; FAO et al., 2015). Crop yield gaps could exist due to many factors, viz: (a) bio-physical and edaphic constraints (biotic/abiotic stress, poor soil fertility and health, high slope and local soil problems); (b) climatic variability and extreme climatic events (drought, flood, hail, heat stress, frost etc.); (c) sub-optimal land and crop management practices (nutrient deficiency or imbalance, poor disease, pest and weed control, non-optimal planting/sowing, inefficient water management etc.); and (d) socio-economical limitations (limited access to financial services, as well as institutional or political constraints, including market access and price) (van Ittersum et al., 2013; Kassie et al., 2014; Beza et al., 2017; van Oort et al., 2017). However, the main reasons for existing yield gaps are sub-optimal management practices associated with nutrient deficiency, together with poor water, disease, pest and weed management. The yield gap has been estimated and reviewed for a variety of crops in different studies at field, national, regional and global scales; for example, in the Global Yield Gap and Water Productivity Atlas (GYGA - www.yieldgap.org) (Boogaard et al., 2013; van Ittersum et al., 2013; FAO et al., 2015; Anderson et al., 2016; Ma et al., 2016; Jain et al., 2017; Fischer and Connor, 2018; Hochman and Horan, 2018; Schils et al., 2018). It should be noted that a full yield gap closure is not always feasible, economically viable or environmentally desirable due to climatic risk, diminishing returns and negative environmental impacts (Lobell et al., 2009; van Ittersum et al., 2013).

A crop ‘genetic yield potential’ is defined as the yield of an ideal or optimal genotype that would capture natural resources efficiently and produce the highest yield in a target environment. Thus, estimation of ‘genetic yield potential’ is based on the possible gain in crop yield that could be achieved through genetic improvements or genotype/germplasm developments, and helps to quantify the scope of potential genetic improvements (Reynolds et al., 2009; Fischer and Edmeades, 2010; Hall and Richards, 2013; Dowla et al., 2018; Senapati et al., 2019a). The important role of *in silico* crop ideotype design in genetic crop improvement for yield gain has been outlined by different researchers (Reynolds et al., 2009; Martre et al., 2015; Ramirez-Villegas et al., 2015; Rötter et al., 2015). The notion of a crop ideotype was first introduced by Donald (1968) as a virtual ‘idealised crop’ in a target environment, which would be expected to produce a greater or optimal quality and quantity of yield when developed as a cultivar. Recently, Senapati et al. (2019a); Senapati and Semenov (2019) estimated wheat ‘genetic yield potential’ by using a crop simulation model and designing crop ideotypes optimised for a target environment, based on optimal physiology constrained by available genetic variation in target traits. They also defined a ‘genetic yield gap’ in a location as the difference between ‘genetic yield potential’ of a crop and yield of a present, well-adapted cultivar under optimal management. Estimating crop ‘genetic yield potential’ and ‘genetic yield gap’ could accelerate crop genetic improvements by quantifying the exploitable gap.

Wheat (*Triticum aestivum* L.) is one of the key staple crops for global food security, providing about 20% of total dietary calories and protein (Shiferaw et al., 2013). Europe is a major wheat producer, contributing around 35% of global wheat production (FAOSTAT, 2019). While the conventional yield potential and yield gap (van Ittersum et al., 2013) have been estimated in Europe (Boogaard et al., 2013; Ma et al., 2016; Schils et al., 2018), the genetic yield potential and genetic yield gap of this important wheat producer are as yet unknown. Estimating wheat genetic yield potential and genetic yield gap in Europe is therefore essential for increasing wheat yields, with the aim of achieving global food security through crop genotype improvements and closing the existing yield gap as much as is feasible under the prevailing resource constraints (Hall and Richards, 2013).

Designing crop ideotypes requires a process-based eco-physiological crop model which (a) is well calibrated and validated locally and (b) has a computational framework for multidimensional optimisation of cultivar traits by utilizing the full parameter space. Designing crop ideotypes also depends on interactions between crop modellers and breeders/geneticists for identifying feasible trait combinations (Martre et al., 2015; Rötter et al., 2015; Tao et al., 2017). In the present study, for ideotype design we used Sirius, a process-based wheat model that has been calibrated and validated in diverse environments across the world, including Europe (Jamieson et al., 2000; Stratonovitch and Semenov, 2010; Wang et al., 2017b; Asseng et al., 2019). Sirius incorporates a powerful ideotype optimisation framework based on an evolutionary search algorithm with self-adaptation (EASA) (Stratonovitch and Semenov, 2010). The main objective of the study was to estimate current wheat genetic yield potential and the genetic yield gap in Europe by designing wheat ideotypes. Wheat ideotypes were optimised for maximum yield in rainfed conditions under current local climates across major wheat growing regions in Europe, based on optimal crop physiology and available genetic variation in target traits.

## 2 Methods

### 2.1 Study sites and current climatic conditions

Thirteen sites across Europe were selected, representing the major and contrasting wheat growing regions in Europe, from Spain in the south to Denmark in the north, and Hungary in the east to the UK in the west (Table S1, Fig. S1). The detailed site characteristics, local current wheat cultivars (cv) and typical sowing dates can be found in Table S1 (Semenov and Shewry, 2011). Detailed characteristics of local wheat cultivars can be found in Table S2. At each study site, 30-years (1981–2010) of daily observed weather data were used for estimating site specific climatic parameters of a stochastic weather generator (*LARS-WG 6.0*) (Semenov and Stratonovitch, 2015). To account for variation in crop yield due to inter-annual climatic variability and climatic extremes, a 100 years of daily weather data at each site were generated by using *LARS-WG 6.0* based on locally estimated climatic parameters, hereafter defined as the ‘current climate’ at individual sites, with a corresponding atmospheric CO_2_ concentration of 364 ppm. The mean annual air temperature, annual precipitation and mean daily global radiation at the study sites under current climate varied from 7.1 to 19.2 °C, 344–801 mm yr^−1^ and 9.7–17.0 MJ m^−2^ day^−1^, respectively (Table S1 and Fig. S1).

### 2.2 Sirius model

Sirius (2018) is a processed-based eco-physiological wheat simulation model with a daily timescale, which includes an optimisation framework for designing crop ideotypes and optimisation of cultivar traits for target environments (Jamieson et al., 1998). The model utilizes the multicore architecture of modern computers, thus reduces substantially the running time for computationally-intensive tasks such as designing ideotypes. The model requires daily weather data, a soil profile description, management information and cultivar description as model inputs. Sirius consists of various sub-models that describe soil, water and nitrogen (N) uptake, photosynthesis and biomass production, crop phenological development, and partitioning of photosynthates into leaf, stem, grain and root etc. Photosynthesis and biomass production are simulated on a daily time-scale as the product of intercepted, photosynthetically active radiation (PAR) and radiation use efficiency (RUE), limited by temperature and water stress. N limitation and abiotic stresses (commonly heat and drought) over the whole crop growing period could accelerate leaf senescence, and thereby reduce photosynthesis and grain yield. In addition to common heat and water stresses, Sirius has recently been improved to simulate the effects of short-term extreme climatic events, such as heat and drought stress around flowering. A short spell of heat and drought stress around flowering could remarkably reduce primary grain setting number (Ji et al., 2010; Onyemaobi et al., 2017), while potential grain size could be reduced by short episodes of heat and drought stress during endosperm development and grain filling (Prasad and Djanaguiraman, 2014). These impacts of short-term, extreme climatic events on grain yield have been incorporated into the Sirius model (Stratonovitch and Semenov, 2015; Senapati et al., 2019b). Table S2 shows important cultivar parameters and traits used in Sirius. Sirius was extensively calibrated and validated for a range of modern wheat cultivars, and has performed well under diverse climatic conditions across Europe and around the world (Jamieson et al., 2000; Stratonovitch and Semenov, 2010; Wang et al., 2017b; Webber et al., 2018; Asseng et al., 2019). A detailed description of the Sirius model can be found elsewhere (Jamieson et al., 1998; Stratonovitch and Semenov, 2015; Senapati et al., 2019b).

### 2.3 Designing wheat ideotypes

An ideotype was defined as a set of Sirius cultivar parameters (traits) that would deliver high yield when optimised in a target environment. A cultivar based on an ideotype and utilizing its optimal combination of trait values would deliver maximum yields for the environment in question. The wheat ideotypes were optimised for grain yield under current climate in rainfed conditions at individual study sites across Europe. Local current wheat cultivars (Avalon, Cartaya, Claire, Creso, Mercia and Thesee) were used as ‘parents’ for designing site-specific wheat ideotypes. A list of the 21 Sirius cultivar parameters is presented in Table S2. Two contrasting ideotypes were designed for rainfed conditions under current climatic conditions: heat and drought sensitive around flowering (iS), and heat and drought tolerant around flowering (iT). Potential primary fertile grain setting number in sensitive wheat genotypes could be reduced even by a short spell of heat and drought stress around flowering due to premature abortion of florets, malfunction and irreversible abortion of male and female reproductive organs and gametophytes, reduced viability of gametophytes, and male and female sterility (Prasad and Djanaguiraman, 2014; Onyemaobi et al., 2017). Tolerance to heat and drought stress around flowering is, therefore, crucial for high yield potential under future climate conditions to maintain greater potential primary grain setting number and grain size (Stratonovitch and Semenov, 2015; Senapati et al., 2019b). First, the two distinct types of ideotype, iS and iT, were set separately as sensitive and tolerant to heat and drought stress around flowering by assigning two different sets of parameters that control sensitivity and tolerance to heat and drought stress at that stage (e.g., HSGNT, HSGNR, DSGNT, DSGNS, DSGNRMax; Table S2) (Stratonovitch and Semenov, 2015; Senapati et al., 2019b). Then, seven cultivar parameters (Table S3) were optimised in both ideotypes independently under current climatic conditions, as described below, while the rest of the parameters remained the same as in the ideotypes' respective parents (Table S2).

### 2.4 Target traits for optimisation

Seven cultivar parameters, related to different wheat cultivar traits, viz canopy structure, phenological development, root water uptake and tolerance to water stress, were selected for optimisation (Table S3) due to their (a) importance in crop yield improvements; (b) large natural genetic variations observed for wheat germplasms; and (c) large potential for improvements through genetic adaptation (Manschadi et al., 2006; Semenov and Stratonovitch, 2013; Christopher et al., 2016). (1) Potential maximum area of flag leaf (A_m__ax_). This is a key trait, influencing crop growth and final grain yield by modifying the rate of canopy expansion and the maximum achievable leaf area, which in turn change the pattern of light interception and transpiration demand (Jamieson et al., 1998; Semenov and Stratonovitch, 2013). A smaller A_m__ax_ would help to avoid drought stress, particularly in dry environments, by reducing transpiration and root water uptake. (2) The ‘stay green’ trait (S_G_). This trait is important in increasing grain yield by delaying leaf senescence after anthesis and maintaining the green leaf area longer for photosynthesis to continue during grain filling (Luche et al., 2015; Christopher et al., 2016). (3) Phyllochron (P_h_: thermal time required for the appearance of successive leaves). (4) Daylength response (P_p_). (5) ‘Duration of grain filling’ (G_f_). This trait controls the rate of phenological development and physiological maturity (Jamieson et al., 1998, 2007). Whereas P_h_ and P_p_ are the major drivers of controlling optimal flowering time, a longer G_f_ will increase grain yield by not only increasing the amount of intercepted radiation during grain filling, but also increase the chance of complete relocation of plant labile carbohydrate into grains. Thus, G_f_ is an important trait for increasing grain yield (Semenov and Stratonovitch, 2013; Soriano et al., 2018). (6) ‘Rate of root water uptake’ (R_u_). This is a key trait affecting temporal patterns and total amount of water uptake (Manschadi et al., 2006). Faster root water uptake could reduce the water stress experienced by plants in anticipation of additional available water later in the season, but may have penalties under terminal drought conditions. In contrast, an alternative strategy of slower root water uptake might increase yield by conserving water for successful completion of the life cycle in water-limited conditions with terminal drought. (7) ‘Maximum acceleration of leaf senescence due to water stress’ (W_ss_). This is a critical trait that controls the increase in the rate of leaf senescence due to water stress under drought (Senapati et al., 2019b). Earlier leaf senescence could reduce grain yield due to reduced cumulative intercepted radiation and premature termination of grain filling. A reduced W_ss_ would reduce leaf senescence and help in survival and tolerance to water stress.

### 2.5 Ideotype optimisation

At each site, both the ideotypes (iS and iT) were optimised independently for maximum grain yield in rainfed conditions under the local current climate. A universal search optimisation method, evolutionary search algorithm with self-adaptation (EASA), was used in Sirius to optimise ideotypes by utilizing the full parameter ranges in a multi-dimensional parameter space with a complex fitness function (Semenov and Terkel, 2003; Stratonovitch and Semenov, 2010). Local wheat cultivars were used as a site-specific initial ‘parent’. At each step of optimisation, 16 new candidate ideotypes were generated from a ‘parent’ by perturbing its cultivar parameters randomly (mutating) within the predefined parameter ranges (Table S3). The parameter ranges were based on cultivar parameters calibrated for modern wheat cultivars, allowing variations corresponding to the existing genetic variation in target traits for wheat germplasm. For each of 16 candidate ideotypes, yield was simulated for 100 years under the current climate. Candidates with a coefficient of variation (CV) of yield exceeding 10% or a harvest index (HI) over 0.64 were removed from the selection process. A CV of less than 10% guarantees high yield stability, whereas the reported upper limit of HI is 0.64 (Foulkes et al., 2011). The candidate with the highest mean yield was selected as a parent for the next step. The optimisation process continued until no further improvement in yield was possible, or parameters converged to an optimal state. To avoid convergence to a local maximum and to explore fully the parameter spaces, we initialized the EASA with multiple ‘parents’. For each site, we used 25 initial parents that were randomly scattered in the parameter space, except one parent that had the same cultivar parameters as the site-specific cultivar. We observed robust convergence of parameters at individual sites; therefore, increasing the number of initial parents further would have had very little benefit. For each of the 25 initial parents, EASA converged to an optimal combination of cultivar parameters; the best candidate was then selected as an optimal ideotype for a selected site.

### 2.6 Simulation setup

Sirius version 2018 (available from https://sites.google.com/view/sirius-wheat) was used. A common medium soil-water profile (Hafren) with a total available water capacity of 177 mm was used at all sites to eliminate site-specific soil effects from the analysis. Sirius was run first for current local wheat cultivars (cv) in rainfed conditions under the current climate to assess present management optimal yields (Y_W_). Then, Sirius was used for designing wheat ideotypes (heat and drought sensitive (iS) or tolerant (iT) around flowering) under the same current climate in rainfed conditions using the same sowing dates as for the current local cultivars. Zhu et al. (2010) indicated that up to 10% more carbon would be assimilated at the current atmospheric CO_2_ level if the Rubisco specificity factor (λ) that represents the discrimination between CO_2_ and O_2_ were optimal. Thus, a 10% increased light use efficiency (LUE) was used for the wheat ideotypes. All simulations were assumed to be water-limited under rainfed conditions and optimally managed, e.g. no nutrient deficiency and no yield losses due to disease, pests or competition with weeds.

### 2.7 Estimation of genetic yield gap

The ‘genetic yield gap’ (Y_iG_) of wheat in water-limited condition (rainfed) under the current climate in a given location was estimated as (Senapati and Semenov, 2019)

Y_iG_ (t ha^-1^) = Y_iW_ – Y_W_

where, Y_iW_ is the water-limited yield (t ha^−1^) of wheat ideotype optimised by Sirius in a given location, representing ‘genetic yield potential’ (Reynolds et al., 2009; Hall and Richards, 2013; Semenov and Stratonovitch, 2013; Martre et al., 2015; Ramirez-Villegas et al., 2015; Rötter et al., 2015; Dowla et al., 2018; Senapati et al., 2019a; Senapati and Semenov, 2019), while Y_W_ is the yield (t ha^−1^) of the current local well-adapted cultivars in rainfed condition under optimal management estimated by using Sirius.

The genetic yield gap was also expressed as proportion (%) of genetic yield potential as

Y_iG_ (%) = (Y_iG_ / Y_iW_) × 100

## 3 Results

### 3.1 Management-optimal rainfed yields of current local wheat cultivars across Europe

**Fig. 1:**
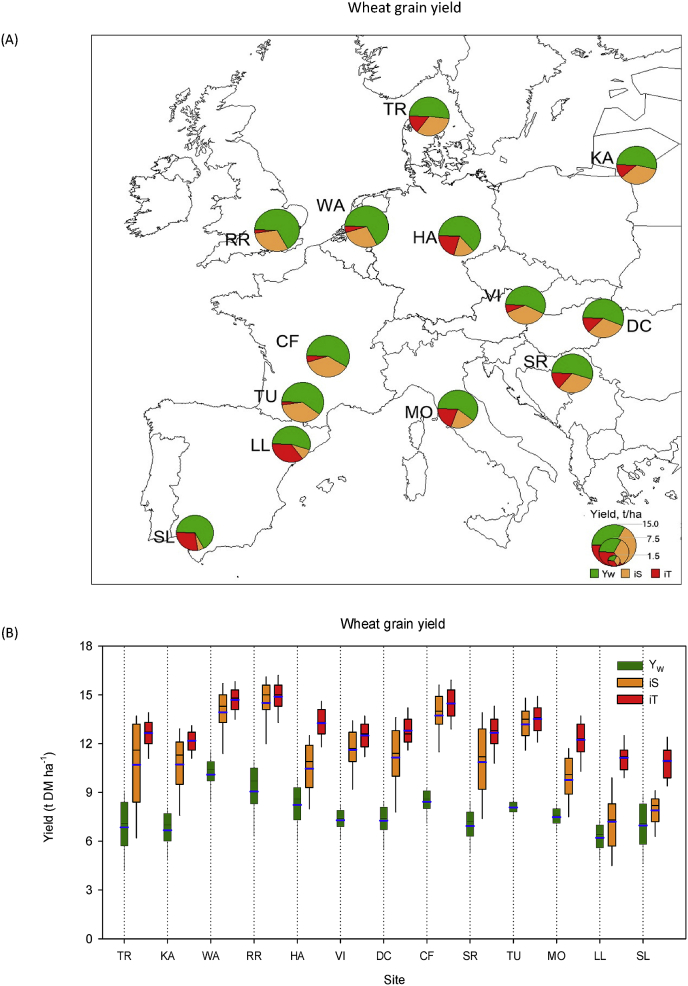
Simulated management optimal grain yield of current local wheat cultivars (cv) under current climate in rainfed condition (Y_W_) at study sites across major wheat growing regions in Europe, and wheat ideotypes designed as heat and drought sensitive (iS) or tolerant (iT) around flowering to achieve high yield potentials. (A) A green sector represents yields for current local wheat cultivars in water limited condition under optimal management (Y_W_), an orange sector shows an increase in yield for iS ideotype compared to Y_W_, and a red sector shows an increase in yield for iT ideotype compared to iS. (B) Green, orange and red coloured box plots represent absolute yields (5-, 25-, 50-, 75- and 95-percentiles) including mean (blue bar) for 100-years under current climate of present local cultivar (Y_W_) and iS and iT ideotypes in rainfed conditions and optimal management. (For interpretation of the references to colour in this figure legend, the reader is referred to the Web version of this article.)

The simulated management optimal grain yields of current well-adapted local wheat cultivars in rainfed condition (Y_W_) under the current climate varied from 6 to 10 t ha^−1^ across Europe, with a mean yield of 7.7 t ha^−1^ (Fig. 1). The highest yield (10 t ha^−1^) was obtained in north-western (NW-) Europe (RR, WA), followed by central-western (CW-) (~8 t ha^−1^) (CF, TU) and central-eastern (CE-) (7–8 t ha^−1^) Europe (HA, VI, DC, SR, MO), whereas yield was lowest (6–7 t ha^−1^) in south-western (SW-) (SL, LL) and north-eastern (NE-) Europe (TR, KA).

### 3.2 Wheat genetic yield potentials in rainfed condition across Europe

Y_iW_ of wheat ideotypes iS and iT varied from 7 to 15 t ha^−1^ and 11–15 t ha^−1^, respectively, across Europe (Fig. 1). Averaged yields of ideotypes iS (11.2 t ha^−1^) and iT (12.9 t ha^−1^) represented 45% (3.5 t ha^−1^) and 69% (5.2 t ha^−1^) greater yields, respectively, than corresponding Y_W_. Over Europe, the mean yield of the iT ideotype was 18% (1.7 t ha^−1^) greater compared to iS. However, the yield difference between iT and iS ideotypes varied widely (from 3 to 55%) at individual sites as described below. Across both the ideotypes, yield potential was highest (13–15 t ha^−1^) in NW and CW Europe, whereas the lowest yield potential (7–11 t ha^−1^) was in SW Europe. Wheat yield potential was intermediate (10–13 t ha^−1^) in NE and CE Europe.

### 3.3 Key traits for optimal wheat genetic yield potentials

**Fig. 2:**
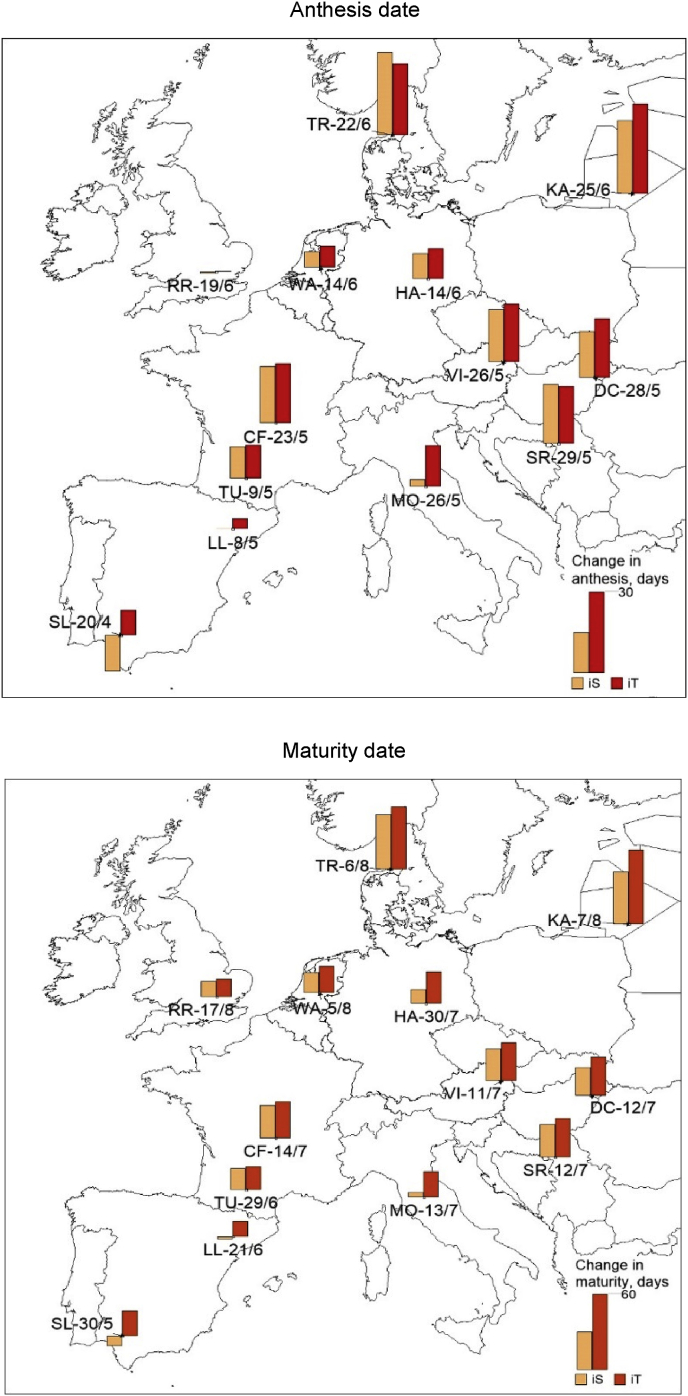
Anthesis and maturity dates of local current wheat cultivars (cv) in rainfed condition under current climate and optimal management at study sites across Europe. Bars represent changes in anthesis and maturity dates of wheat ideotypes viz. sensitive iS (orange) and tolerant iT (red) compared to local wheat cultivars under optimal managements. (For interpretation of the references to colour in this figure legend, the reader is referred to the Web version of this article.)

**Fig. 3:**
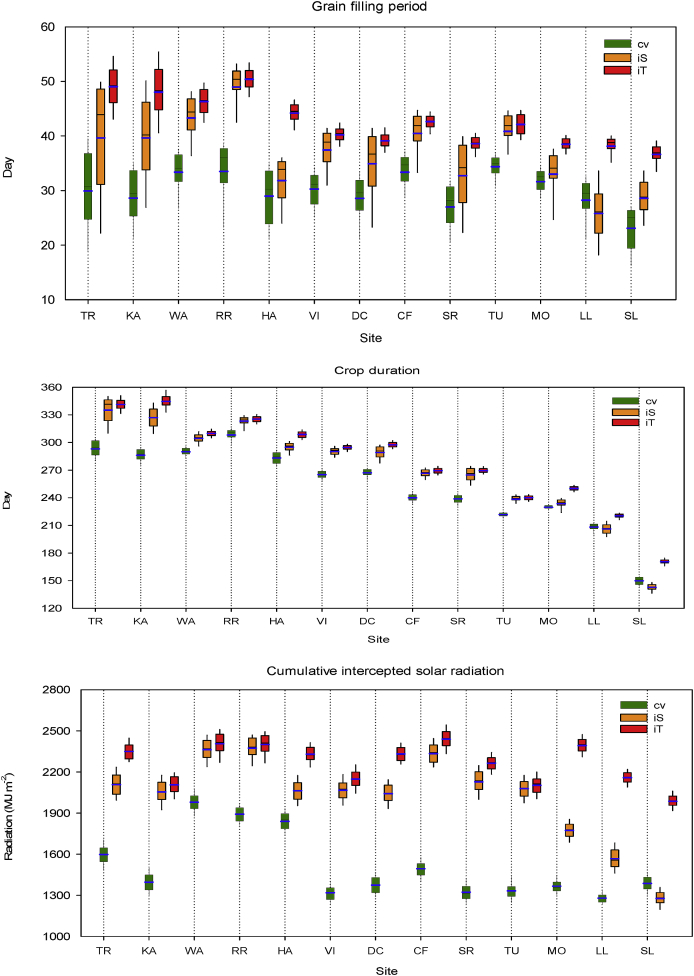
Grain filling period, crop duration and cumulative intercepted solar radiation of current local wheat cultivars (cv, green) in rainfed condition under current climate and optimal management at study sites across Europe, and wheat ideotypes designed as heat and drought sensitive (iS, orange) and tolerant (iT, red) around flowering to achieve high yield potentials. (For interpretation of the references to colour in this figure legend, the reader is referred to the Web version of this article.)

Table S4 shows the designs of optimal wheat ideotypes to achieve high genetic yield potentials at study sites across major wheat growing regions in Europe. Overall, these optimal ideotype traits were found to be far removed from the corresponding traits of local cultivars. The highest yield benefit (3–4 t ha^-1^; ~39–55%) of heat and drought tolerance around flowering compared with heat and drought sensitivity was obtained in SW Europe, followed by NE and CE Europe (0.9–3 t ha^-1^; ~ 8–27%), whereas a minimum or almost no benefit was found in NW and CW Europe (0.3–0.8 t ha^-1^; ~ 3–6%) (Fig. 1). Thus, the results indicated heat and drought tolerance around flowering to be a key trait for adaptation to achieve optimal wheat genetic yield potential in Europe. Averaged over idiotypes, an improved canopy structure, in terms of flag leaf area (A_max_ ~77%) and stay-green trait (S_G_ ~91%), was identified as important for high yield potentials in Europe, as was optimal phenology, in terms of anthesis time, grain filling period and physiological maturity (crop duration) (Fig. 2, Fig. 3). Optimal anthesis time was highly localised with wide site variation. The mean optimal anthesis time over Europe was 12 and 16 days later than the local current cultivars (205-days after sowing, DAS) for iS and iT ideotypes, respectively. But, an early anthesis time was found to be beneficial for the iS ideotype, particularly at Seville, Spain. On average, an extension to the grain filling period of 7 days (iS ideotype) to 12 days (iT ideotype) over current wheat cultivars (30-days) was found as optimal. Similarly, a mean maturity date of 18 (iS) to 28 (iT) days later than for present local cultivars (253 DAS) was optimal, with the exception that early maturity was helpful for optimal yield of the iS ideotype in SW Europe. The mean improved rates of root water uptake for both wheat ideotypes compared to present cultivars (R_u_ ~73%) indicated that an improved root system would be beneficial for achieving high potential yields. In contrast, reduced leaf senescence due to water stress (W_ss_ ~11%) would be helpful for improved photosynthesis and grain yields. Overall, a better optimal canopy architecture, phenology and reduced leaf senescence were obtained for the iT ideotype than the iS, but a greater root water uptake rate was found for iS (Fig. 2, Fig. 3, Table S4).

### 3.4 Wheat genetic yield gap in Europe

**Fig. 4:**
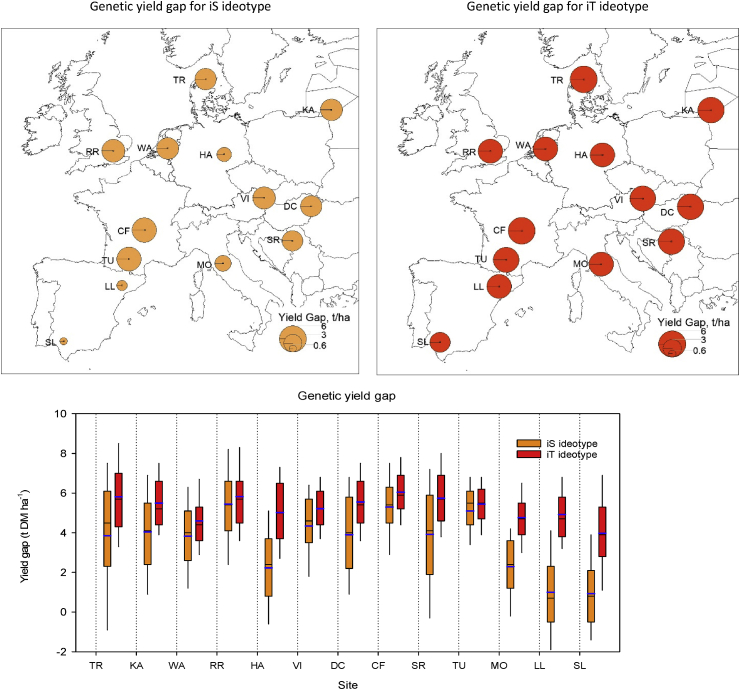
Wheat genetic yield gap (Y_iG_) in rainfed condition under current climate at study sites across major wheat growing regions in Europe.

The current wheat genetic yield gap (Y_iG_) under rainfed conditions ranged between 0.6 and 6 t ha^-1^ across ideotypes in major wheat growing regions in Europe, representing 8–48% of wheat genetic yield potential (Fig. 4). However, Y_iG_ varied for different regions in Europe and the ideotype types, iT and iS. The mean genetic yield gap overall for Europe was 30% (3.5 t ha^-1^) for the iS ideotype and 40% (5.2 t ha^-1^) for the iT. For the iS ideotype, the absolute yield gap was highest (4–5 t ha^-1^) in NW and CW Europe, whereas it was lowest (≤1 t ha^-1^) in SW Europe. The yield gap was intermediate (2–4 t ha^-1^) in NE and CE Europe. In contrast, variation in the yield gap for the iT ideotype was relatively small between sites; indeed, the yield gap was almost equal (5–6 t ha^-1^) at all the sites except for one site in SW Europe (SL; Y_iG_ ~3.6 t ha^-1^). The absolute yield gap as well as the percent yield gap at individual sites and over Europe as a whole were greater for the iT ideotype than iS: The mean absolute yield gap over Europe was 49% (1.7 t ha^-1^) greater for iT ideotype than iS, while the highest yield gap difference between the iT and iS ideotypes was in SW-Europe (3–4 t ha^-1^), followed by NE and CE Europe (1–3 t ha^-1^), whereas the difference was minimum (<1 t ha^-1^) in NW and CW Europe.

## 4 Discussion

The present study is the first of its kind to estimate wheat genetic yield potential (Y_iW_) and genetic yield gap (Y_iG_) under the current climate in water limited conditions across Europe by optimising ideotypes in local environmental conditions. Ideotype optimisation was based on state-of-the-art knowledge in crop physiology (Table S2) and available genetic variation in target traits (Table S3). Several recent studies have designed wheat ideotypes for maximizing yield potentials under current and future climatic conditions (Sylvester-Bradley et al., 2012; Tao et al., 2017; Wang et al., 2019; Xin and Tao, 2019). However, they did not consider available genetic variation in crop germplasm or optimise cultivar traits to find a global optimum.

Various studies have reported the yields of current adapted wheat cultivars in rainfed conditions under optimal managements (Y_W_) as 4–13 t ha^-1^, with associated yield gaps of 0.2–6.9 t ha^-1^ across Europe, with the highest and lowest potential yield and yield gaps in NW and SW Europe, respectively, due to these regions having the most and least favourable climatic conditions (Boogaard et al., 2013; Ma et al., 2016; Schils et al., 2018). It is worth noting that the estimated Y_W_ in the present study of 6–10 t ha^-1^ across Europe falls well within the range reported by the other European studies mentioned above.

Optimised wheat ideotypes developed in the current study represent the best possible crop genetic adaptations at selected local environments, constrained by the available genetic variations in target traits. Thus, optimal wheat ideotypes can make the best possible use of local environmental conditions and represent the ‘genetic yield potential’ (Donald, 1968; Reynolds et al., 2009; Hall and Richards, 2013; Semenov and Stratonovitch, 2013; Lopes et al., 2015; Martre et al., 2015; Ramirez-Villegas et al., 2015; Rötter et al., 2015; Dowla et al., 2018; Senapati et al., 2019a). We identified the key traits and their optimal combinations for genetic adaptation (Table S4) to achieve genetic yield potential viz. heat and drought tolerance around flowering, optimal canopy structure, optimal crop phenology, improved root water uptake and tolerance to drought stress.

A short spell of heat or drought stress around flowering can drastically reduce primary grain setting number and final yield in wheat (Ji et al., 2010; Prasad and Djanaguiraman, 2014; Onyemaobi et al., 2017), and tolerance of heat and drought stresses around flowering has been identified as a key trait for achieving high wheat yield, particularly in hot and dry regions (Stratonovitch and Semenov, 2015; Senapati et al., 2019b). The highest yield benefits due to heat and drought tolerance around flowering were in SW, NE and CE Europe, with the lowest in NW and CW Europe, due to the higher and lower heat and drought stresses in those regions, respectively (Fig. S2).

An optimal canopy architecture is needed for maximum interception of solar radiation, photosynthesis and ultimately high yield potential (Reynolds et al., 2009; Hawkesford et al., 2013). A greater A_max_ resulted in larger leaf area, which contributed to increased interception of solar radiation and yield for both ideotypes (Fig. 3 and Fig. S3). Improvement in the stay-green trait enabled wheat ideotypes to maintain green leaf area longer after anthesis, enabling photosynthesis to continue during grain filling, resulting in higher yield potential (Christopher et al., 2016).

Optimal flowering time is a crucial trait for adaptation, as it determines the delicate balance between achieving high biomass (carbon source) and maximum primary grain setting (carbon sink) at anthesis, and influences the climatic conditions (e.g., cooler temperature and high radiation) that prevail during grain filling (Kamran et al., 2014). Early flowering might help wheat to ‘escape’ from heat and drought stresses at anthesis (Shavrukov et al., 2017), for example as found particularly in SW Europe for heat and drought sensitive ideotypes in the present study. In contrast, late flowering could help to maximize pre-anthesis biomass production, which would ultimately contribute to higher yield, as in the case of most of the current study regions in Europe. Extending the grain filling period is another key trait for improving grain yield by increasing intercepted radiation, the production of photosynthates for direct grain filling, and the translocation of pre-stored labile carbohydrates from the vegetative tissues into the grains (Semenov and Stratonovitch, 2013; Soriano et al., 2018). A late maturity and corresponding extended crop duration could be beneficial to increase cumulative intercepted solar radiation, photosynthesis and final grain yield (Fig. 1, Fig. 2, Fig. 3). Higher thermal requirements in terms of P_h_ and G_f_ (Table S4) could help to delay anthesis and maturity, also improving yield (Wang et al., 2017a; Asseng et al., 2019).

A larger, deeper and more efficient root system, corresponding to an improved ‘rate of root water uptake’ (R_u_), is often recommended for avoidance, survival or tolerance of severe drought and heat stress (Manschadi et al., 2006). A reduced ‘leaf senescence acceleration due to water stress’ (W_ss_) linked to an overall drought tolerance during the whole crop season is an important trait for reducing leaf senescence and increasing photosynthesis and yield under water stress conditions (Jamieson et al., 1998; Lawless et al., 2005).

Site and regional variations in wheat genetic yield potential and yield gap across Europe could be explained by variations in the local optimal wheat physiology, determined by local environmental conditions. For example, the highest yield potential in NW and CW Europe are due to the most favourable climatic conditions (air temperature, precipitation etc.) with minimum heat and drought stresses around flowering. This helps the better optimisation of cultivar traits, such as delayed flowering time, longer grain filling period and crop duration, resulting in greater intercepted solar radiation and yield (Fig. 1, Fig. 2, Fig. 3, S1 and S2; Table S1). In contrast, in SW, NE and CE Europe, yield potentials are constrained by high temperature and heat stress and/or low precipitation and drought stress, which result in early anthesis and maturity, with shorter grain filling and crop duration, and ultimately lower cumulative intercepted radiation and grain yields.

The highest genetic yield potential and genetic yield gap were in NW and CW Europe, with intermediate genetic yield potential and genetic yield gap in CE and NE Europe, indicating that current local wheat cultivars are not yet optimised for yield (Table S4) and that considerable genetic yield gaps still exist in those regions despite intensive breeding efforts. Thus, wheat yield could be improved substantially in those regions by using optimal cultivars. On the other hand, the lowest yield potential and yield gap were in the lowest wheat productive region (SW), showing that present cultivars are near optimal under the local environmental conditions in that region, and further yield improvement is unlikely unless heat and drought tolerance around flowering can be achieved.

A vast available genetic variation exists for different yield-improving traits in wheat germplasm (see ranges in Table S3). In the last few decades, substantial progress has been achieved in identifying QTL and underpinning genes associated with key traits for wheat adaptation; for example, heat and drought tolerance (Acuna-Galindo et al., 2015), flag leaf area (Liu et al., 2018), stay green (Christopher et al., 2018), flowering time and maturity (Vrn, Ppd and Eps) (Langer et al., 2014) and root architecture (VRN1, Rht-B1b, qTALRO-B1) (Cao et al., 2014). Diverse allelic variation (Ppd-A1 and Ppd-B1) has also been found for grain filling duration (Royo et al., 2016). Commercial bread wheat genotypes (Onyemaobi et al., 2017), transgenic lines (Pradhan et al., 2012) and durum wheat landraces (Lopes et al., 2015) are available with different degrees of tolerance to heat and drought stress. Near-isogenic lines of wheat with different allelic variation (Ppd-1) in the control of flowering time have also been developed (Perez-Gianmarco et al., 2018), while genotypes with a diverse range in thermal requirements exist in global germplasm collections (Prajapat and Saxena, 2018). At the same time, modern plant breeding technology has advanced, including gene mapping and molecular marker-assisted breeding, genomics-assisted breeding and gene editing (e.g. using CRISPR-Cas9) (Breseghello and Coelho, 2013). A cautious averaged time frame of past genetic improvement in a target crop trait varies from 5 to 20 years (Hall and Richards, 2013). However, the above-mentioned wheat genetic resources and recent technological advances in breeding provide opportunities to develop improved wheat genotypes much faster for target environments. The estimated large unexploited genetic yield gap for wheat in Europe along with identified key traits should motivate plant scientists and breeders to strive for further genetic improvements and develop better genotypes for yield gain in wheat.

In conclusion, a large wheat genetic yield potential of 11–13 t ha^-1^ was estimated across Europe under the current climate in water limited conditions, depending on the sensitivity or tolerance of wheat ideotypes to heat and drought stresses around flowering. Despite intensive wheat breeding efforts, current local cultivars were found to be far from their optimum, and a large genetic yield gap (3.5–5.2 t ha^-1^; ~30–40% of genetic yield potential) still exists in Europe. Heat and drought tolerance around flowering, optimal canopy structure and phenology, improved root water uptake and reduced leaf senescence due to water stress were identified as key traits for genetic adaptation to achieve genetic yield potentials. Closing a large unexploited genetic yield gap in Europe would contribute towards genetic yield improvement and underpin global food security.

## Declaration of competing interest

The authors declare no conflicts of interest.

## References

[bib1] Acuna-Galindo M.A., Mason R.E., Subramanian N.K., Hays D.B. (2015). Meta-analysis of wheat QTL regions associated with adaptation to drought and heat stress. Crop Sci..

[bib2] Anderson W., Johansen C., Siddique K.H.M. (2016). Addressing the yield gap in rainfed crops: a review. Agron. Sustain. Dev..

[bib3] Asseng S., Martre P., Maiorano A., Rotter R.P., O'Leary G.J., Fitzgerald G.J., Girousse C., Motzo R., Giunta F., Babar M.A., Reynolds M.P., Kheir A.M.S., Thorburn P.J., Waha K., Ruane A.C., Aggarwal P.K., Ahmed M., Balkovic J., Basso B., Biernath C., Bindi M., Cammarano D., Challinor A.J., De Sanctis G., Dumont B., Rezaei E.E., Fereres E., Ferrise R., Garcia-Vila M., Gayler S., Gao Y.J., Horan H., Hoogenboom G., Izaurralde R.C., Jabloun M., Jones C.D., Kassie B.T., Kersebaum K.C., Klein C., Koehler A.K., Liu B., Minoli S., San Martin M.M., Muller C., Kumar S.N., Nendel C., Olesen J.E., Palosuo T., Porter J.R., Priesack E., Ripoche D., Semenov M.A., Stockle C., Stratonovitch P., Streck T., Supit I., Tao F.L., Van der Velde M., Wallach D., Wang E.L., Webber H., Wolf J., Xiao L.J., Zhang Z., Zhao Z.G., Zhu Y., Ewert F. (2019). Climate change impact and adaptation for wheat protein. Glob. Chang. Biol..

[bib4] Beza E., Silva J.V., Kooistra L., Reidsma P. (2017). Review of yield gap explaining factors and opportunities for alternative data collection approaches. Eur. J. Agron..

[bib5] Boogaard H., Wolf J., Supit I., Niemeyer S., van Ittersum M. (2013). A regional implementation of WOFOST for calculating yield gaps of autumn-sown wheat across the European Union. Field Crop. Res..

[bib6] Breseghello F., Coelho A.S.G. (2013). Traditional and modern plant breeding methods with examples in Rice (Oryza sativa L.). J. Agric. Food Chem..

[bib7] Cao P., Ren Y.Z., Zhang K.P., Teng W., Zhao X.Q., Dong Z.Y., Liu X., Qin H.J., Li Z.S., Wang D.W., Tong Y.P. (2014). Further genetic analysis of a major quantitative trait locus controlling root length and related traits in common wheat. Mol. Breed..

[bib8] Cassman K.G. (2012). What do we need to know about global food security?. Glob. Food Secur.-Agric.Policy.

[bib9] Christopher J.T., Christopher M.J., Borrell A.K., Fletcher S., Chenu K. (2016). Stay-green traits to improve wheat adaptation in well-watered and water-limited environments. J. Exp. Bot..

[bib10] Christopher M., Chenu K., Jennings R., Fletcher S., Butler D., Borrell A., Christopher J. (2018). QTL for stay-green traits in wheat in well-watered and water-limited environments. Field Crop. Res..

[bib11] Donald C.M. (1968). The breeding of crop ideotypes. Euphytica.

[bib12] Dowla M., Edwards I., O'Hara G., Islam S., Ma W. (2018). Developing wheat for improved yield and adaptation under a changing climate: Optimization of a few key genes. Engineering.

[bib13] FAO (2009). Global Agriculture towards 2050. How to Feed the World in 2050.

[bib14] FAO, Sadras V.O., Cassman K.G.G., Grassini P., Hall A.J., Bastiaanssen W.G.M., Laborte A.G., Milne A.E., Sileshi G., Steduto P. (2015).

[bib15] FAO, IFAD, UNICEF, WFP, WHO (2018). The State of Food Security and Nutrition in the World. Building Climate Resilience for Food Security and Nutrition. The State of the World.

[bib16] FAOSTAT (2019). FAOSTAT Crop Database. Food and Agriculture Organisation of the United Nations. http://www.fao.org/faostat/en/#data/QC.

[bib17] Fischer R.A., Connor D.J. (2018). Issues for cropping and agricultural science in the next 20 years. Field Crop. Res..

[bib18] Fischer R.A.T., Edmeades G.O. (2010). Breeding and cereal yield progress. Crop Sci..

[bib19] Foley J.A., Ramankutty N., Brauman K.A., Cassidy E.S., Gerber J.S., Johnston M., Mueller N.D., O'Connell C., Ray D.K., West P.C., Balzer C., Bennett E.M., Carpenter S.R., Hill J., Monfreda C., Polasky S., Rockstrom J., Sheehan J., Siebert S., Tilman D., Zaks D.P.M. (2011). Solutions for a cultivated planet. Nature.

[bib20] Foulkes M.J., Slafer G.A., Davies W.J., Berry P.M., Sylvester-Bradley R., Martre P., Calderini D.F., Griffiths S., Reynolds M.P. (2011). Raising yield potential of wheat. III. Optimizing partitioning to grain while maintaining lodging resistance. J. Exp. Bot..

[bib21] Godfray H.C.J., Beddington J.R., Crute I.R., Haddad L., Lawrence D., Muir J.F., Pretty J., Robinson S., Thomas S.M., Toulmin C. (2010). Food security: the challenge of feeding 9 billion people. Science.

[bib22] Hall A.J., Richards R.A. (2013). Prognosis for genetic improvement of yield potential and water-limited yield of major grain crops. Field Crop. Res..

[bib23] Hawkesford M.J., Araus J.L., Park R., Calderini D., Miralles D., Shen T., Zhang J., Parry M.A.J. (2013). Prospects of doubling global wheat yields. Food Energy Secur.

[bib24] Hochman Z., Horan H. (2018). Causes of wheat yield gaps and opportunities to advance the water-limited yield frontier in Australia. Field Crop. Res..

[bib25] Jain M., Singh B., Srivastava A.A.K., Malik R.K., McDonald A.J., Lobell D.B. (2017). Using satellite data to identify the causes of and potential solutions for yield gaps in India's Wheat Belt. Environ. Res. Lett..

[bib26] Jamieson P.D., Berntsen J., Ewert F., Kimball B.A., Olesen J.E., Pinter P.J., Porter J.R., Semenov M.A. (2000). Modelling CO_2_ effects on wheat with varying nitrogen supplies. Agric. Ecosyst. Environ..

[bib27] Jamieson P.D., Brooking I.R., Semenov M.A., MeMaster G.S., White J.W., Porter J.R. (2007). Reconciling alternative models of phenological development in winter wheat. Field Crop. Res..

[bib28] Jamieson P.D., Semenov M.A., Brooking I.R., Francis G.S. (1998). Sirius: a mechanistic model of wheat response to environmental variation. Eur. J. Agron..

[bib29] Ji X., Shiran B., Wan J., Lewis D.C., Jenkins C.L.D., Condon A.G., Richards R.A., Dolferus R. (2010). Importance of pre-anthesis anther sink strength for maintenance of grain number during reproductive stage water stress in wheat. Plant Cell Environ..

[bib30] Kamran A., Iqbal M., Spaner D. (2014). Flowering time in wheat (Triticum aestivum L.): a key factor for global adaptability. Euphytica.

[bib31] Kassie B.T., Van Ittersum M.K., Hengsdijk H., Asseng S., Wolf J., Rotter R.P. (2014). Climate-induced yield variability and yield gaps of maize (Zea mays L.) in the Central Rift Valley of Ethiopia. Field Crop. Res..

[bib32] Langer S.M., Longinand C.F.H., Wurschum T. (2014). Flowering time control in European winter wheat. Front. Plant Sci..

[bib33] Lawless C., Semenov M.A., Jamieson P.D. (2005). A wheat canopy model linking leaf area and phenology. Eur. J. Agron..

[bib34] Liu K.Y., Xu H., Liu G., Guan P.F., Zhou X.Y., Peng H.R., Yao Y.Y., Ni Z.F., Sun Q.X., Du J.K. (2018). QTL mapping of flag leaf-related traits in wheat (Triticum aestivum L.). Theor. Appl. Genet..

[bib35] Lobell D.B., Cassman K.G., Field C.B. (2009). Crop yield gaps: their importance, magnitudes, and causes. Annu. Rev. Environ. Resour..

[bib36] Lopes M.S., El-Basyoni I., Baenziger P.S., Singh S., Royo C., Ozbek K., Aktas H., Ozer E., Ozdemir F., Manickavelu A., Ban T., Vikram P. (2015). Exploiting genetic diversity from landraces in wheat breeding for adaptation to climate change. J. Exp. Bot..

[bib37] Luche H.D., da Silva J.A.G., da Maia L.C., de Oliveira A.C. (2015). Stay-green: a potentiality in plant breeding. Ciência Rural..

[bib38] Ma S.X., Churkina G., Gessler A., Wieland R., Bellocchi G. (2016). Yield gap of winter wheat in Europe and sensitivity of potential yield to climate factors. Clim. Res..

[bib39] Manschadi A.M., Christopher J., deVoil P., Hammer G.L. (2006). The role of root architectural traits in adaptation of wheat to water-limited environments. Funct. Plant Biol..

[bib40] Martre P., Quilot-Turion B., Luquet D., Memmah M.-M.O.-S., Chenu K., Debaeke P., Sadras V.O., Calderini D.F. (2015). Model-assisted phenotyping and ideotype design. Crop Physiology: Applications for Genetic Improvement and Agronomy.

[bib41] Onyemaobi I., Liu H., Siddique K.H.M., Yan G.J. (2017). Both male and female malfunction contributes to yield reduction under water stress during meiosis in bread wheat. Front. Plant Sci..

[bib42] Perez-Gianmarco T.I., Slafer G.A., Gonzalez F.G. (2018). Wheat pre-anthesis development as affected by photoperiod sensitivity genes (Ppd-1) under contrasting photoperiods. Funct. Plant Biol..

[bib43] Pradhan G.P., Prasad P.V.V., Fritz A.K., Kirkham M.B., Gill B.S. (2012). Effects of drought and high temperature stress on synthetic hexaploid wheat. Funct. Plant Biol..

[bib44] Prajapat A.L., Saxena R. (2018). Thermal requirements of wheat (Triticum aestivum L.) cultivars under different growing environments. Int. J. Chem. Stud..

[bib45] Prasad P.V.V., Djanaguiraman M. (2014). Response of floret fertility and individual grain weight of wheat to high temperature stress: sensitive stages and thresholds for temperature and duration. Funct. Plant Biol..

[bib46] Ramirez-Villegas J., Watson J., Challinor A.J. (2015). Identifying traits for genotypic adaptation using crop models. J. Exp. Bot..

[bib47] Reynolds M., Foulkes M.J., Slafer G.A., Berry P., Parry M.A.J., Snape J.W., Angus W.J. (2009). Raising yield potential in wheat. J. Exp. Bot..

[bib48] Rötter R.P., Tao F., Höhn J.G., Palosuo T. (2015). Use of crop simulation modelling to aid ideotype design of future cereal cultivars. J. Exp. Bot..

[bib49] Royo C., Dreisigacker S., Alfaro C., Ammar K., Villegas D. (2016). Effect of Ppd-1 genes on durum wheat flowering time and grain filling duration in a wide range of latitudes. J. Agric. Sci..

[bib50] Schils R., Olesen J.E., Kersebaum K.-C., Rijk B., Oberforster M., Kalyada V., Khitrykau M., Gobin A., Kirchev H., Manolova V., Manolov I., Trnka M., Hlavinka P., Palosuo T., Peltonen-Sainio P., Jauhiainen L., Lorgeou J., Marrou H., Danalatos N., Archontoulis S., Fodor N., Spink J., Roggero P.P., Bassu S., Pulina A., Seehusen T., Uhlen A.K., Żyłowska K., Nieróbca A., Kozyra J., Silva J.V., Maçãs B.M., Coutinho J., Ion V., Takáč J., Mínguez M.I., Eckersten H., Levy L., Herrera J.M., Hiltbrunner J., Kryvobok O., Kryvoshein O., Sylvester-Bradley R., Kindred D., Topp C.F.E., Boogaard H., de Groot H., Lesschen J.P., van Bussel L., Wolf J., Zijlstra M., van Loon M.P., van Ittersum M.K. (2018). Cereal yield gaps across Europe. Eur. J. Agron..

[bib51] Semenov M.A., Shewry P.R. (2011). Modelling predicts that heat stress, not drought, will increase vulnerability of wheat in Europe. Sci. Rep..

[bib52] Semenov M.A., Stratonovitch P. (2013). Designing high-yielding wheat ideotypes for a changing climate. Food Energy Secur.

[bib53] Semenov M.A., Stratonovitch P. (2015). Adapting wheat ideotypes for climate change: accounting for uncertainties in CMIP5 climate projections. Clim. Res..

[bib54] Semenov M.A., Terkel D.A. (2003). Analysis of convergence of an evolutionary algorithm with self-adaptation using a stochastic Lyapunov function. Evol. Comput..

[bib55] Senapati N., Brown H.E., Semenov M.A. (2019). Raising genetic yield potential in high productive countries: designing wheat ideotypes under climate change. Agric. For. Meteorol..

[bib56] Senapati N., Semenov M.A. (2019). Assessing yield gap in high productive countries by designing wheat ideotypes. Sci. Rep..

[bib57] Senapati N., Stratonovitch P., Paul M.J., Semenov M.A. (2019). Drought tolerance during reproductive development is important for increasing wheat yield potential under climate change in Europe. J. Exp. Bot..

[bib58] Shavrukov Y., Kurishbayev A., Jatayev S., Shvidchenko V., Zotova L., Koekemoer F., de Groot S., Soole K., Langridge P. (2017). Early flowering as a drought escape mechanism in plants: how can it aid wheat production?. Front. Plant Sci..

[bib59] Shiferaw B., Smale M., Braun H.J., Duveiller E., Reynolds M., Muricho G. (2013). Crops that feed the world 10. Past successes and future challenges to the role played by wheat in global food security. Food Secur.

[bib60] Soriano J.M., Villegas D., Sorrells M.E., Royo C. (2018). Durum wheat landraces from east and west regions of the Mediterranean basin are genetically distinct for yield components and phenology. Front. Plant Sci..

[bib61] Stratonovitch P., Semenov M.A. (2010). Calibration of a crop simulation model using an evolutionary algorithm with self-adaptation. Proc. Social Behavior. Sci..

[bib62] Stratonovitch P., Semenov M.A. (2015). Heat tolerance around flowering in wheat identified as a key trait for increased yield potential in Europe under climate change. J. Exp. Bot..

[bib63] Sylvester-Bradley R., Riffkin P., O'Leary G. (2012). Designing resource-efficient ideotypes for new cropping conditions: wheat (Triticum aestivum L.) in the high rainfall zone of southern Australia. Field Crop. Res..

[bib64] Tao F.L., Rotter R.P., Palosuo T., Diaz-Ambrona C.G.H., Minguez M.I., Semenov M.A., Kersebaum K.C., Nendel C., Cammarano D., Hoffmann H., Ewert F., Dambreville A., Martre P., Rodriguez L., Ruiz-Ramos M., Gaiser T., Hohn J.G., Salo T., Ferrise R., Bindi M., Schulman A.H. (2017). Designing future barley ideotypes using a crop model ensemble. Eur. J. Agron..

[bib65] UN (2017). World Population Prospects: the 2017 Revision. https://population.un.org/wpp/.

[bib66] van Ittersum M.K., Cassman K.G., Grassini P., Wolf J., Tittonell P., Hochman Z. (2013). Yield gap analysis with local to global relevance-A review. Field Crop. Res..

[bib67] van Oort A.J., Saito K., Dieng I., Grassini P., Cassman K.G., van Ittersum M.K. (2017). Can yield gap analysis be used to inform R & D prioritisation?. Glob. Food Secur.-Agric. Policy.

[bib68] Wang B., Feng P.Y., Chen C., Liu D.L., Waters C., Yu Q. (2019). Designing wheat ideotypes to cope with future changing climate in South-Eastern Australia. Agric. Syst..

[bib69] Wang B., Liu D.L., Asseng S., Macadam I., Yu Q. (2017). Modelling wheat yield change under CO2 increase, heat and water stress in relation to plant available water capacity in eastern Australia. Eur. J. Agron..

[bib70] Wang E., Martre P., Zhao Z., Ewert F., Maiorano A., Rötter R.P., Kimball B.A., Ottman M.J., Wall G.W., White J.W., Reynolds M.P., Alderman P.D., Aggarwal P.K., Anothai J., Basso B., Biernath C., Cammarano D., Challinor A.J., De Sanctis G., Doltra J., Dumont B., Fereres E., Garcia-Vila M., Gayler S., Hoogenboom G., Hunt L.A., Izaurralde R.C., Jabloun M., Jones C.D., Kersebaum K.C., Koehler A.-K., Liu L., Müller C., Naresh Kumar S., Nendel C., O'Leary G., Olesen J.E., Palosuo T., Priesack E., Eyshi Rezaei E., Ripoche D., Ruane A.C., Semenov M.A., Shcherbak I., Stöckle C., Stratonovitch P., Streck T., Supit I., Tao F., Thorburn P., Waha K., Wallach D., Wang Z., Wolf J., Zhu Y., Asseng S. (2017). The uncertainty of crop yield projections is reduced by improved temperature response functions. Native Plants.

[bib71] Webber H., Ewert F., Olesen J.E., Muller C., Fronzek S., Ruane A.C., Bourgault M., Martre P., Ababaei B., Bindi M., Ferrise R., Finger R., Fodor N., Gabaldon-Leal C., Gaiser T., Jabloun M., Kersebaum K.C., Lizaso J.I., Lorite I.J., Manceau L., Moriondo M., Nendel C., Rodriguez A., Ruiz-Ramos M., Semenov M.A., Siebert S., Stella T., Stratonovitch P., Trombi G., Wallach D. (2018). Diverging importance of drought stress for maize and winter wheat in Europe. Nat. Commun..

[bib72] Xin Y., Tao F.L. (2019). Optimizing genotype-environment-management interactions to enhance productivity and eco-efficiency for wheat-maize rotation in the North China Plain. Sci. Total Environ..

[bib73] Zhu X.G., Long S.P., Ort D.R. (2010). Improving photosynthetic efficiency for greater yield. Annu. Rev. Plant Biol..

