# Supplementary material for: Large genetic yield potential and genetic yield gap estimated for wheat in Europe
Source: Glob Food Sec. 2020 Mar;24:100340. doi: 10.1016/j.gfs.2019.100340 (PMC7063691; doi:10.1016/j.gfs.2019.100340)
Supplement: Multimedia component 1 [file mmc1.pdf]

Large genetic yield potential and genetic yield gap estimated for wheat in Europe

Supplementary Information

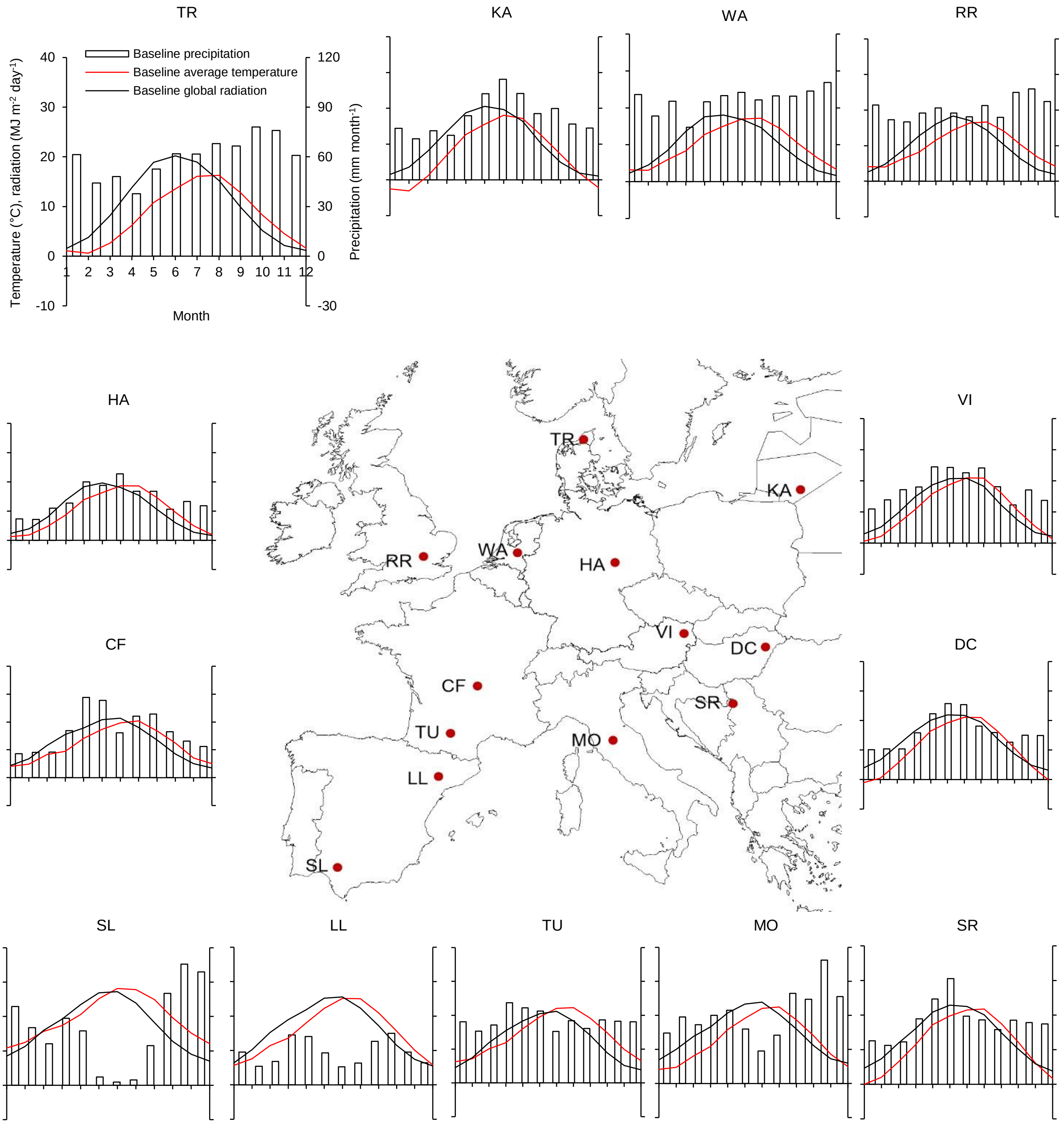

Supplementary Fig. S1. Locations of 13 selected study sites, representing major wheat growing regions across Europe. The current climate viz. average air temperature, mean monthly precipitation and mean daily global solar radiation.

Heat stress index (HSI95)

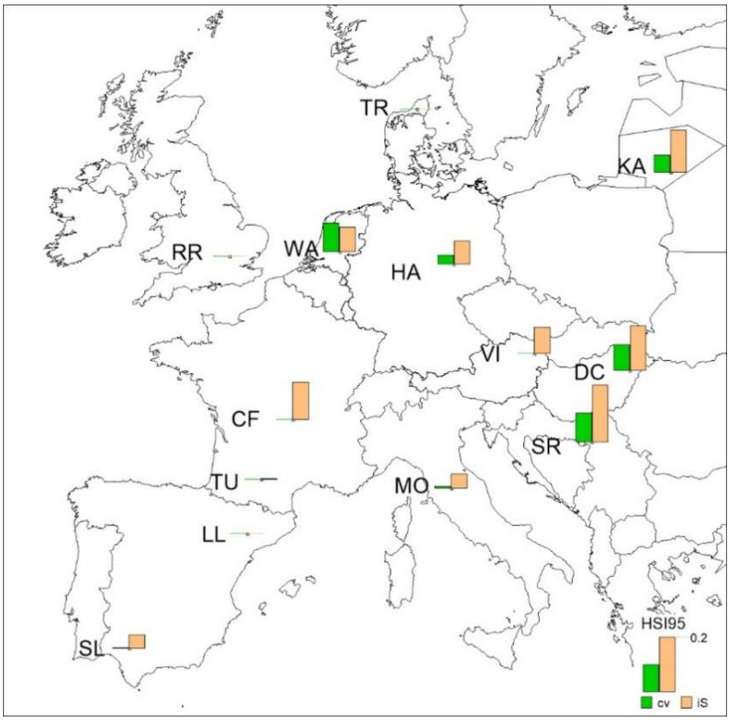

Drought stress index (DSI95)

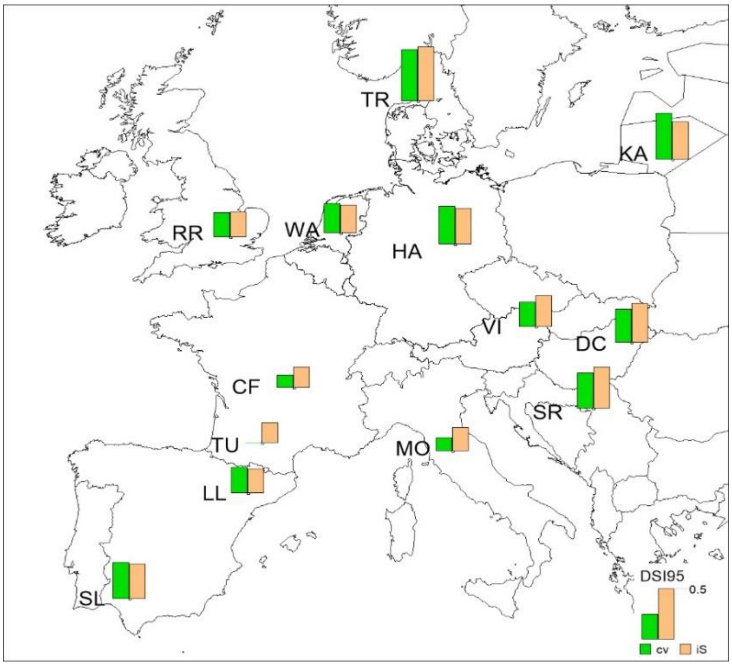

Supplementary Fig. S2. 95-percentile of heat stress index (HSI95) and 95-percentile of drought stress index (DSI95) of local wheat cultivars (cv, green) in rainfed condition under current climate and optimal management at study sites across Europe, and heat and drought sensitive around flowering wheat ideotypes (iS, orange) optimised to achieve high yield potentials. HSI = (1 – Y_{wh}/Y_w), DSI = (1 – Y_{wd}/Y_w), where Y_w is water limited yield, Y_{wh} is water limited yield of wheat sensitive to heat stress around flowering, and Y_{wd} is water limited yield of wheat sensitive to drought stress around flowering.

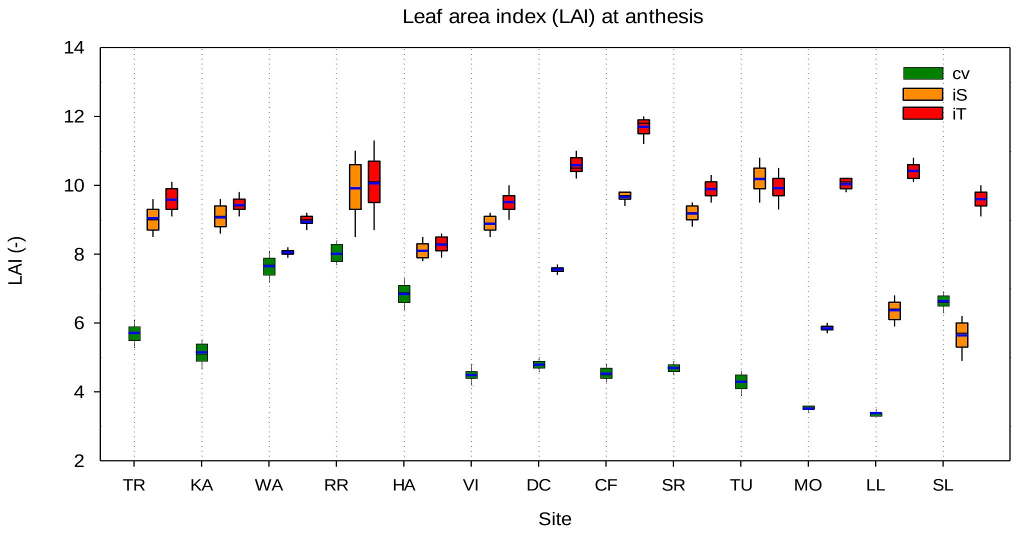

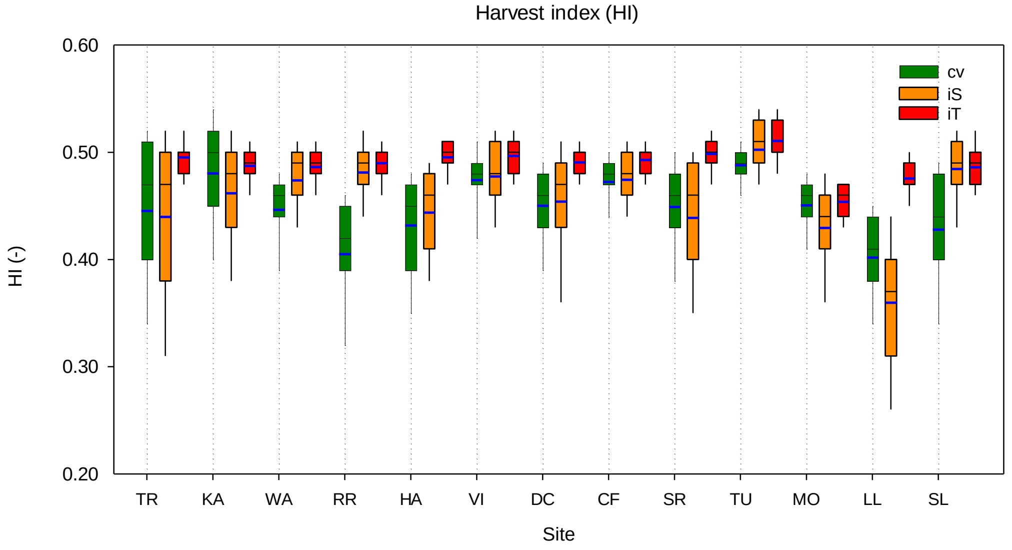

Supplementary Fig. S3. Simulated leaf area index (LAI) at anthesis and harvest index (HI) of local wheat cultivar (cv, green) in rainfed condition under current climate and optimal management at study sites across Europe, and wheat ideotypes designed as heat and drought sensitive (iS, orange) and tolerant (iT, red) around flowering to achieve high yield potentials.

Supplementary Table S1. Study sites across major wheat growing regions in Europe

No.	ID	Site	Country	Latitude (º)	Longit-ude (º)	Average air temperat-ure (ºC)	Precipita-tion (mm yr^{-1})	Radiation (MJ m^{-2} day^{-1})	Local wheat cultivar (cv)	Sowing date of local wheat cultivar
1	TR	Tylstrup	Denmark	57.20	9.90	7.9	717	9.9	Avalon	18-Oct
2	KA	Kaunas	Lithuania	54.88	23.83	7.1	644	10.0	Avalon	25-Oct
3	WA	Wageningen	Netherlands	51.97	5.67	9.7	801	9.7	Claire	20-Oct
4	RR	Rothamsted	UK	51.80	-0.35	9.7	735	9.7	Mercia	10-Oct
5	HA	Halle	Germany	51.51	11.95	9.6	507	10.0	Claire	20-Oct
6	VI	Vienna	Austria	48.23	16.35	10.8	651	11.4	Thesee	20-Oct
7	DC	Debrecen	Hungary	47.60	21.60	10.4	591	12.7	Thesee	18-Oct
8	CF	Clermont-Ferrand	France	45.80	3.10	11.6	606	12.2	Thesee	15-Nov
9	SR	Sremska	Serbia	45.00	19.51	11.5	654	13.4	Thesee	15-Nov
10	TU	Toulouse	France	43.62	1.38	13.8	666	12.4	Thesee	20-Nov
11	MO	Montagnano	Italy	43.30	11.80	12.8	738	14.7	Creso	25-Nov
12	LL	Lleida	Spain	41.63	0.60	15.1	344	15.6	Creso	25-Nov
13	SL	Seville	Spain	37.42	-5.88	19.2	595	17.0	Cartaya	30-Dec

Supplementary Table S2. Description of the cultivar parameters (traits) of the local current wheat cultivars (cv) at study sites across major wheat growing regions in Europe.

No.	Parameters	Symbol	Unit	Value					
				Avalon	Cartaya	Claire	Creso	Mercia	Thesee
1	Phyllochron	P_h	ºC day	90.0	105.0	110.0	90.0	107.0	94.0
2	Day length response	P_p	Leaf h^{-1}day length	0.65	0.20	0.50	0.60	0.53	0.4
3	Thermal time from sowing to emergence	TT_{SOWEM}	ºC day	150.0	150.0	150.0	160.0	150.0	175.0
4	Thermal time from anthesis to beginning of grain fill	TT_{ANBGF}	ºC day	50.0	100.0	100.0	100.0	160.0	100.0
5	Thermal time from beginning of grain fill to end of grain fill	TT_{BGFEGF}	ºC day	650.0	550.0	650.0	650.0	650.0	650.0
6	Thermal time from end of grain fill to harvest maturity	TT_{EGFMAT}	ºC day	200.0	200.0	200.0	200.0	200.0	200.0
7	Maximum area of flag leaf	A_{Max}	m^2 leaf m^{-2} soil	0.0065	0.0065	0.007	0.003	0.0075	0.004
8	Minimum possible leaf number	L_{Min}	-	8.55	8.50	8.0	8.50	8.0	8.0
9	Absolute maximum leaf number	L_{Max}	-	24.0	24.0	18.0	24.0	24.0	18.0
10	Response of vernalisation rate to temperature	*VAI*	Day^{-1}°C	0.0012	0	0.0012	0.0015	0.0012	0.0012
11	Vernalisation rate at 0°C	*VBEE*	Day^{-1}	0.015	0	0.012	0.012	0.011	0.012
12	Heat stress grain number reduction threshold temperature	*HSGNT*	ºC	30.0	30.0	30.0	30.0	30.0	30.0
13	Heat stress grain number reduction rate	*HSGNR*	$ºC^{-1}$	0.04	0.04	0.04	0.04	0.04	0.04
14	Drought stress grain number reduction stress threshold	*DSGNT*	-	0.90	0.90	0.90	0.90	0.90	0.90
15	Drought stress grain number reduction stress saturation	*DSGNS*	-	0.30	0.30	0.30	0.30	0.30	0.30
16	Maximum drought stress grain number reduction	*DSGNRMax*	-	0.20	0.20	0.20	0.20	0.20	0.20
17	Maximum potential grain weight	*MaxGW*	g	0.045	0.045	0.045	0.045	0.045	0.045
18	Grain number per g DM ear	*GNEar*	g^{-1}	100	100	100	100	100	100
19	Stay green	S_G	-	0.50	0.50	0.50	0.50	0.50	0.50
20	Rate coefficient of root water uptake from the root bottom	R_u	-	0.03	0.03	0.03	0.03	0.03	0.03
21	Maximum leaf senescence acceleration factor due to water stress	W_{ss}	-	1.27	1.27	1.27	1.27	1.27	1.27

Supplementary Table S3. Cultivar parameters (traits) used for designing wheat ideotypes in rainfed condition under current climate and genetic variation observed in those parameters (traits).

Parameters	Symbol	Unit	Range used in model optimization	Genetic variation	Reference
Canopy structure					
Maximum area of flag leaf	A_{Max}	m^2 leaf m^{-2} soil	0.005–0.01	≤40%	Fischer et al., 1998; Shearman et al., 2005
Stay green	S_G	-	0.00–1.50		
Canopy Phenology					
Phyllochron	P_h	ºC day	80.0–140.0	≤20%	Ishag et al., 1998; Mosaad et al., 1995
Day length response	P_p	Leaf h^{-1}day length	0.05–0.90	9.74–107.40†	Kosner and Zurkova, 1996
Duration of grain filling	G_f	ºC day	500–900	≤40%	Akkaya et al., 2006; Charmet et al., 2005; Robert et al., 2001
Root water uptake					
Rate coefficient of root water uptake from the root bottom	R_u	-	0.01–0.07	Large variation	Asseng et al., 1998; Manschadi et al., 2006
Drought tolerance					
Maximum leaf senescence acceleration factor due to water stress	W_{ss}	-	1.0–1.7		

†Varietal difference in number of days till heading under long- and short-day conditions found between 9.74 and 107.40 in a photoperiodic response experiment (Kosner & Zurkova 1996)[5]

References

Akkaya, A., Dokuyucu, T., Kara, R., Akçura, M., 2006. Harmonization ratio of post- to pre-anthesis durations by thermal times for durum wheat cultivars in a Mediterranean environment. Eur. J. Agron. 24, 404-408. https://doi.org/10.1016/j.eja.2005.10.005.

Asseng, S., Ritchie, J.T., Smucker, A.J.M., Robertson, M.J., 1998. Root growth and water uptake during water deficit and recovering in wheat. Plant Soil 201, 265-273. https://doi.org/10.1023/a:1004317523264.

Charmet, G., Robert, N., Branlard, G., Linossier, L., Martre, P., Triboï, E., 2005. Genetic analysis of dry matter and nitrogen accumulation and protein composition in wheat kernels. Theor. Appl. Genet. 111, 540-550. https://doi.org/10.1007/s00122-005-2045-1.

Fischer, R.A., Rees, D., Sayre, K.D., Lu, Z.M., Condon, A.G., Saavedra, A.L., 1998. Wheat yield progress associated with higher stomatal conductance and photosynthetic rate, and cooler canopies. Crop Sci. 38, 1467-1475. https://doi.org/10.2135/cropsci1998.0011183X003800060011x.

Ishag, H.M., Mohamed, B.A., Ishag, K.H., 1998. Leaf development of spring wheat cultivars in an irrigated heat-stressed environment. Field Crop Res. 58, 167-175. https://doi.org/10.1016/S0378-4290(98)00092-6.

Kosner, J., Zurkova, D., 1996. Photoperiodic response and its relation to earliness in wheat. Euphytica 89, 59-64. https://doi.org/10.1007/BF00015719.

Manschadi, A.M., Christopher, J., deVoil, P., Hammer, G.L., 2006. The role of root architectural traits in adaptation of wheat to water-limited environments. Funct. Plant Biol. 33, 823-837. https://doi.org/10.1071/FP06055.

Mosaad, M.G., Ortizferrara, G., Mahalakshmi, V., Fischer, R.A., 1995. Phyllochron response to vernalization and photoperiod in spring wheat. Crop Sci. 35, 168-171. https://doi.org/10.2135/cropsci1995.0011183X003500010031x.

Robert, N., Berard, P., Hennequet, C., 2001. Dry matter and nitrogen accumulation in wheat kernel. Genetic variation in rate and duration of grain filling [Triticum aestivum L.]. J. Genet. Breed. 55, 297-305.

Shearman, V.J., Sylvester-Bradley, R., Scott, R.K., Foulkes, M.J., 2005. Physiological processes associated with wheat yield progress in the UK. Crop Sci. 45, 175-185. https://doi.org/10.2135/cropsci2005.0175.

Supplementary Table S4. Cultivar parameters (traits) of local current wheat cultivars (cv) at study sites across major wheat growing regions in Europe, and wheat ideotypes designed as heat and drought sensitive (iS) and tolerant (iT) around flowering to achieve high yield potentials in rainfed condition under current climate.

Site	Cultivar/ ideotype	Cultivar parameter/trait						
		A	S_G	P_h	P_p	G_f	R_u	W_{ss}
		(m^2 leaf m^{-2} soil)	(-)	(ºC day)	(Leaf h^{-1}day length)	(ºC day)	(%)	(-)
		Local current wheat cultivar (cv)						
Tylstrup, Denmark (TR)	Avalon	0.0065	0.500	90.0	0.650	650.0	0.030	1.270
Kaunas, Lithuania (KA)	Avalon	0.0065	0.500	90.0	0.650	650.0	0.030	1.270
Wageningen, Netherlands (WA)	Claire	0.0070	0.500	110.0	0.500	650.0	0.030	1.270
Rothamsted, UK (RR)	Mercia	0.0075	0.500	107.0	0.530	650.0	0.030	1.270
Halle Germany (HA)	Claire	0.0070	0.500	110.0	0.500	650.0	0.030	1.270
Vienna, Austria (VI)	Thesee	0.0040	0.500	94.0	0.400	650.0	0.030	1.270
Debrecen, Hungary (DC)	Thesee	0.0040	0.500	94.0	0.400	650.0	0.030	1.270
Clermont-Ferrand, France (CF)	Thesee	0.0040	0.500	94.0	0.400	650.0	0.030	1.270
Sremska, Serbia (SR)	Thesee	0.0040	0.500	94.0	0.400	650.0	0.030	1.270
Toulouse, France (TU)	Thesee	0.0040	0.500	94.0	0.400	650.0	0.030	1.270
Montagnano, Italy (MO)	Creso	0.0030	0.500	90.0	0.600	650.0	0.030	1.270
Lleida, Spain (LL)	Creso	0.0030	0.500	90.0	0.600	650.0	0.030	1.270
Seville, Spain (SL)	Cartaya	0.0065	0.500	105.0	0.200	550.0	0.030	1.270
		Wheat ideotype optimised as sensitive to heat and drought stresses around flowering (iS)						
Tylstrup, Denmark (TR)	Ideotype	0.0081	1.090	138.9	0.459	816.1	0.030	1.330
Kaunas, Lithuania (KA)	Ideotype	0.0100	1.200	129.6	0.662	895.4	0.062	1.291
Wageningen, Netherlands (WA)	Ideotype	0.0064	0.860	140.0	0.072	849.2	0.055	1.010
Rothamsted, UK (RR)	Ideotype	0.0100	1.210	139.2	0.050	900.0	0.067	1.021
Halle Germany (HA)	Ideotype	0.0075	1.310	127.5	0.391	680.2	0.055	1.173
Vienna, Austria (VI)	Ideotype	0.0079	0.610	134.1	0.133	882.4	0.062	1.108
Debrecen, Hungary (DC)	Ideotype	0.0060	1.500	131.1	0.064	900.0	0.058	1.125
Clermont-Ferrand, France (CF)	Ideotype	0.0078	1.170	138.4	0.052	897.8	0.062	1.228
Sremska, Serbia (SR)	Ideotype	0.0081	0.650	139.5	0.050	900.0	0.063	1.164
Toulouse, France (TU)	Ideotype	0.0100	0.770	130.3	0.052	900.0	0.054	1.216
Montagnano, Italy (MO)	Ideotype	0.0050	1.120	101.7	0.336	794.7	0.065	1.205
Lleida, Spain (LL)	Ideotype	0.0055	1.060	106.9	0.166	830.9	0.048	1.673
Seville, Spain (SL)	Ideotype	0.0060	0.680	89.5	0.120	731.7	0.070	1.014
		Wheat Ideotype optimised as tolerant to heat and drought stress around flowering (iT)						
Tylstrup, Denmark (TR)	Ideotype	0.0094	1.430	135.1	0.101	900.0	0.037	1.262
Kaunas, Lithuania (KA)	Ideotype	0.0092	0.230	138.7	0.152	886.2	0.058	1.030
Wageningen, Netherlands (WA)	Ideotype	0.0072	0.530	140.0	0.126	900.0	0.069	1.006
Rothamsted, UK (RR)	Ideotype	0.0100	1.170	140.0	0.055	900.0	0.063	1.000
Halle Germany (HA)	Ideotype	0.0070	1.310	140.0	0.050	898.2	0.058	1.188
Vienna, Austria (VI)	Ideotype	0.0086	0.260	139.5	0.073	900.0	0.061	1.000
Debrecen, Hungary (DC)	Ideotype	0.0100	0.960	140.0	0.094	899.7	0.035	1.000
Clermont-Ferrand, France (CF)	Ideotype	0.0100	1.070	140.0	0.064	899.9	0.046	1.170
Sremska, Serbia (SR)	Ideotype	0.0094	0.950	138.8	0.050	900.0	0.026	1.047
Toulouse, France (TU)	Ideotype	0.0094	0.950	131.0	0.050	899.8	0.070	1.216
Montagnano, Italy (MO)	Ideotype	0.0082	1.200	131.2	0.072	900.0	0.030	1.000
Lleida, Spain (LL)	Ideotype	0.0095	0.450	119.3	0.050	900.0	0.027	1.000
Seville, Spain (SL)	Ideotype	0.0099	1.020	126.3	0.050	900.0	0.021	1.002

A : Maximum area of flag leaf
S_G : Stay green
P_h : Phyllochron
P_p : Day length response
G_f : Duration of grain filling
R_u : Rate coefficient of root water uptake from the root bottom
W_{ss} : Maximum leaf senescence acceleration factor due to water stress
